# Diagnosing Parkinson Disease Through Facial Expression Recognition: Video Analysis

**DOI:** 10.2196/18697

**Published:** 2020-07-10

**Authors:** Bo Jin, Yue Qu, Liang Zhang, Zhan Gao

**Affiliations:** 1 Dalian University of Technology Dalian China; 2 Dongbei University of Finance and Economics Dalian China; 3 Beijing Haoyisheng Cloud Hospital Management Technology Ltd Beijing China

**Keywords:** Parkinson disease, face landmarks, machine learning, artificial intelligence

## Abstract

**Background:**

The number of patients with neurological diseases is currently increasing annually, which presents tremendous challenges for both patients and doctors. With the advent of advanced information technology, digital medical care is gradually changing the medical ecology. Numerous people are exploring new ways to receive a consultation, track their diseases, and receive rehabilitation training in more convenient and efficient ways. In this paper, we explore the use of facial expression recognition via artificial intelligence to diagnose a typical neurological system disease, Parkinson disease (PD).

**Objective:**

This study proposes methods to diagnose PD through facial expression recognition.

**Methods:**

We collected videos of facial expressions of people with PD and matched controls. We used relative coordinates and positional jitter to extract facial expression features (facial expression amplitude and shaking of small facial muscle groups) from the key points returned by Face++. Algorithms from traditional machine learning and advanced deep learning were utilized to diagnose PD.

**Results:**

The experimental results showed our models can achieve outstanding facial expression recognition ability for PD diagnosis. Applying a long short-term model neural network to the positions of the key features, precision and F1 values of 86% and 75%, respectively, can be reached. Further, utilizing a support vector machine algorithm for the facial expression amplitude features and shaking of the small facial muscle groups, an F1 value of 99% can be achieved.

**Conclusions:**

This study contributes to the digital diagnosis of PD based on facial expression recognition. The disease diagnosis model was validated through our experiment. The results can help doctors understand the real-time dynamics of the disease and even conduct remote diagnosis.

## Introduction

The population overall is currently aging. While an aging population represents the triumph of medical and social advances over disease, it also presents daunting challenges. Age is a crucial parameter for the occurrence, development, and diagnosis of diseases. As age increases, the central nervous system’s morphology, metabolism, and function undergo different degrees of decline, which results in certain neurological diseases [1]. One typical example is Parkinson disease (PD), which is caused by a decrease in dopamine secretion. PD, also known as tremor palsy, is a common neurodegenerative disease; the manifestations are mainly bradykinesia, myotonia, resting tremors, and unstable posture [2]. Clinical symptoms may also include nonmotor symptoms such as olfactory function decline, constipation, and depression. As a progressive disease, the various symptoms, both motor and nonmotor, become more serious as the disease course develops, and various complications, such as the “on-off” phenomenon, dyskinesia, and decline in drug efficacy, often occur in the later stages. Patients with serious illness may be plagued by balance disorders, frozen gait, falls, and speech disorders, resulting in an inability to take care of themselves and a decrease in quality of life [3]. Famous people like Xiaoping Deng, Jingrun Chen, Jin Ba, and Muhammad Ali have all been deeply affected by PD. Hence, an accurate diagnosis and medication that works immediately are important.

In recent years, with the development of computer vision technology, facial image recognition has been used for disease diagnosis. In 2017, the National Human Genome Research Institute developed facial diagnosis software to identify whether a child has DiGeorge syndrome [4]. It is a very rare disease, with a pediatric incidence rate ranging from 1/6000 to 1/3000 worldwide. With such a wide range of morbidity, disease diagnosis can be difficult. However, all children with DiGeorge syndrome have clinically obvious facial features, which were utilized by the National Human Genome Research Institute for disease diagnosis. In 2016, FDNA Inc developed the Face2Gene system, which can help doctors diagnose genetic diseases via facial recognition [5]. Some relatively rare genetic diseases can even be discovered by comparing photos of patients with healthy people. This helps patients detect their disease and obtain treatment in a relatively short time.

For PD, the disease-specific facial expressions of patients have attracted researchers’ attention. In the 1860s, Charcot first described the characteristics of “masked face” in patients with PD [6]. Based on years of research, “mask face” is considered one of the common symptoms of PD. Its symptoms involve a faceless and binocular gaze [7]. Facial expressions in humans are expressed by the superficial muscles of the face. These muscles are called the “facial muscles” or “expression muscles.” Smiles and other facial expressions of patients with PD often appear to be unresponsive and have significantly smaller amplitude. The time it takes to form an expression is also extended. To many patients’ families, the “mask face” symptom causes issues because it is an expression of sullenness. Therefore, exploring a diagnostic method using facial feature point recognition is promising. Note that detection of a speech disorder in patients with PD, which is also a noninvasive diagnostic method, has been shown to be effective [8,9]. Hence, facial expression recognition can be combined with speech disorder recognition to obtain a more comprehensive, multidirectional, noninvasive, remote diagnosis.

Several researchers have utilized machine learning and computer vision technology to explore the inner relationship in the “mask face.” Bandini et al [10] proposed an automatic method in 2017 to analyze videos of facial expressions in patients with PD. They extracted the average distances between facial key points using face tracing. Then, they found that patients with PD have much smaller distances of facial movement. In contrast to video-based research, Rajnoha et al [11] designed an automatic detection method based on static facial images using convolutional neural network models. However, the best achieved accuracy was only 67.33%. In 2019, Langevin et al [12] designed the Parkinson's Analysis with Remote Kinetic-tasks framework to analyze PD characteristics, finding that facial features and motion features in the Movement Disorder Society Unified Parkinson’s Disease Rating Scale (MDS-UPDRS) could be extracted from videos.

In order to explore facial expressions in humans and test whether they are accompanied by tremors, we need to extract the key points of the face. In this paper, we investigated the ability to diagnose PD by recognizing changes in key points of the face in a short video. At present, to the best of our knowledge, we are the first to utilize sequential changes in key points of the face to diagnose PD.

## Methods

### Data Collection

People with PD usually suffer from loss of facial expression on both sides of the face. Moreover, their eye movement is reduced, like wearing a mask. This symptom is usually called the “mask face” [13]. Motion retardation is one of the main symptoms of PD. When facial symptoms appear, movement in facial expressions is significantly reduced, accompanied by local tremor symptoms of the small tendon group. When patients with PD try to smile or form other expressions, the facial muscles move slowly and often show excessive expressions. Based on the main facial expression symptoms of patients with PD, we recorded the smiles of patients with PD. Each video was 5 seconds long, and we recorded 2-3 videos per patient. The expression of a smile can be used to distinguish a patient with PD from someone without PD by comparing the magnitude of the expression and trembling of the small muscles of the face.

**Figure 1:**
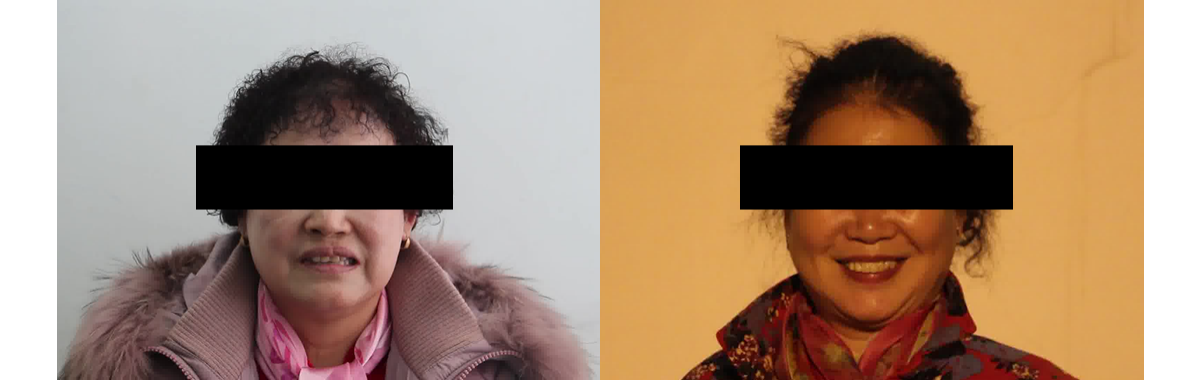
Smiles of a patient with Parkinson disease (left) and person without Parkinson disease (right).

Patients with PD at the First Affiliated Hospital of Dalian Medical University were recruited and provided video data. Patients provided written informed consent before collecting data. Data for the control group, or people without PD, were randomly collected from senior people who liked to exercise frequently. Finally, we collected data from 64 senior citizens, including 33 people with PD. Each person recorded a smile expression 3 times. The length of every video was 5 seconds. After filtering out the videos that could not be used, we finally collected 176 records. The label is the subject status (ie, whether he or she had PD), as shown in Figure 1.

#### Acquisition Equipment

In order to make the captured video clear enough and not affect the experimental results due to human jitter, the videos were captured using a Canon 700D camera placed on a standard tripod.

#### Collection Plan

In the process of recording facial expressions, we showed emoticons, which were printed on photos, to the patients with PD and asked them to imitate the emoticons. This data collection process aimed to explore the vibration of the small muscle groups of the face to distinguish patients with PD from people without PD. The underlying reason for asking the participants to imitate an emoticon in a photo was to prevent the subjective emotions of the patients with PD from affecting the data collection. At the same time, it guaranteed consistency of data collection and ensured that each patient’s understanding of a smile or anger was identical. The data collection process was as follows. First, we recorded the participant’s gender, age, and history of other neurological diseases. Then, the subjects filled out the video data collection registration form, with full awareness of the PD detection research project. Table 1 displays an example of the information collected via the registration form. Third, the emoticon photos were given to the subjects, and they were asked to imitate them. We collected 3 videos of smiling facial expressions, each of which lasted for 5 seconds. After all the recordings were completed, the videos were classified into 2 classes: patients with PD and people without PD. Different folders were sorted according to the patient ID.

**Table 1:** Example data collected using the registration form to collect data via video of patients with Parkinson disease.

Patient Number	Age (years)	Gender	Confirmed	Other neurological disease	Length of disease (month)	Date of collection
1	60	Male	Yes	No	10	11/13/2017
2	55	Male	Yes	No	24	11/13/2017
3	60	Male	Yes	No	10	11/13/2017
4	63	Female	Yes	No	14	11/13/2017

#### Converting Video to Images

To obtain the participant’s facial information, we split the recorded video into individual frames that were extracted directly from the video every 0.1 seconds. In this experiment, we used ffmpeg to implement this function. ffmpeg is a set of open source computer programs that convert digital audio and video into streams [14]. In practice, processing multiple videos in multiple folders involves the files’ operation commands. We leveraged a subprocess module in the python programming environment that encapsulates the running terminal commands, which perfectly met our needs. The subprocess package mainly executes external commands and programs and uses the function *subprocess.call*() to call external commands. The video was converted into several images according to a preset time interval and stored in the specified folder for analysis.

### Data Preprocessing

Face++ is a well-known service for face recognition and facial landmark detection. There are many interesting applications for using Face++, such as inferring the demographics of social media users from profile pictures [15]. We chose Face++ because of its outstanding ability to localize facial landmarks. Based on previous literature and market research on key points of faces, we found that the Face++ interface can provide 106 coordinate points to create very accurate faces at this stage.

**Figure 2:**
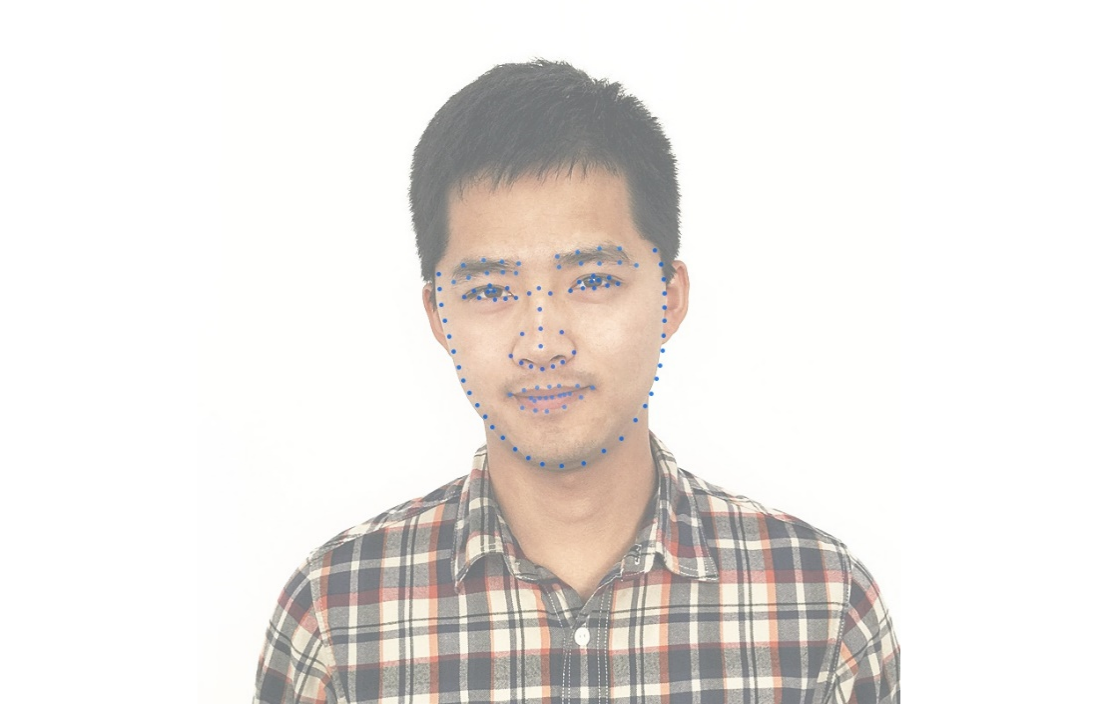
Face key points (n=106) returned by the Face++ interface.

Users simply log in to the Face++ official website and apply for an API key. By calling the “Detect API” interface while using the “POST” method and setting the *return_landmark* parameter value to 2, the system will return 106 key points of the human face, as shown in Figure 2. These 106 points cover most of the key points of a human’s face, including the facial contour, eyebrows, eyes, nose, and mouth that can describe almost all facial expressions. The returned coordinates are numbers using pixels as the unit. The top left corner of the image is used as the origin point.

The facial muscles of patients with PD tend to be more rigid than those of people without PD, which causes a smaller facial expression range. We obtained the magnitude of the patient’s expression by calculating the coordinate positions’ range. However, during the video recording process, we found that although the video equipment is stable, it is difficult to ensure the participant, especially a patient with PD, does not move. To capture a relatively accurate magnitude of the facial expression, we converted the absolute coordinates into relative coordinates. Through the variation of the relative coordinates, the magnitude of the change in the facial expression of the patient can be reflected, and the error caused by postural changes of the body can be avoided.

**Figure 3:**
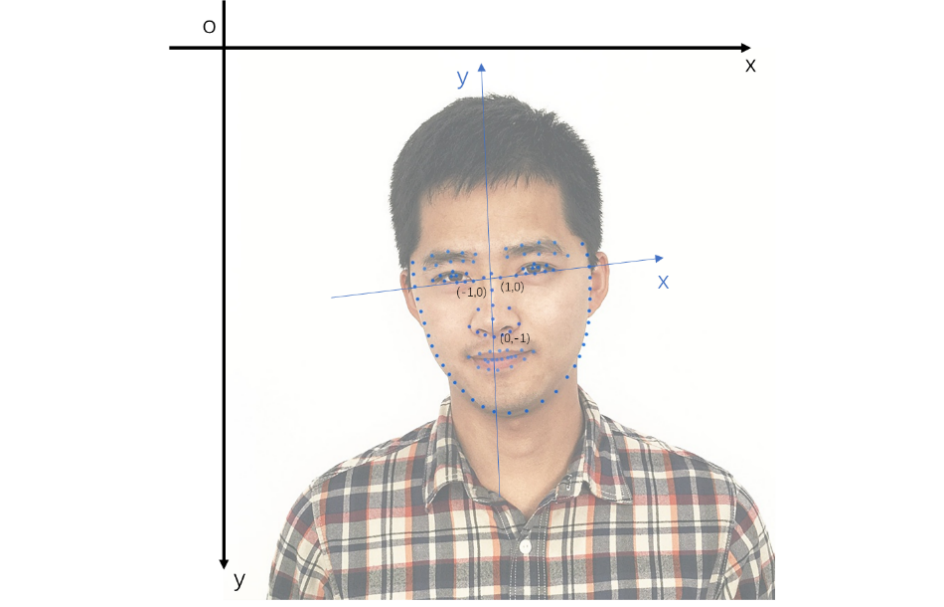
Relative face coordinate system, where the Cartesian, or absolute, coordinate system is represented by the black coordinate system, which was used to record the position of pixels in the image, and the non-Cartesian, or relative, coordinate system is represented by the blue coordinate system, which was used to record the relative position of key points on the face.

To transform absolute coordinates into relative coordinates, we used the midpoint between the inside corners of the eyes as the origin of the coordinate (0,0). Then, we set the line connecting the inside corners of the eye as the x axis and the line connecting the nose and the origin as the y axis. A non-Cartesian coordinate system could then be created. As for the units, the coordinates of the inside corners of both eyes were quantized as (–1,0) and (1,0), and the coordinates of the nose were quantized as (0,–1), as shown in Figure 3. The black coordinate system was used to record the position of the pixels in the image, so we called this the absolute coordinate system. The blue coordinate system represents the relative coordinate system.

**Figure 4:**
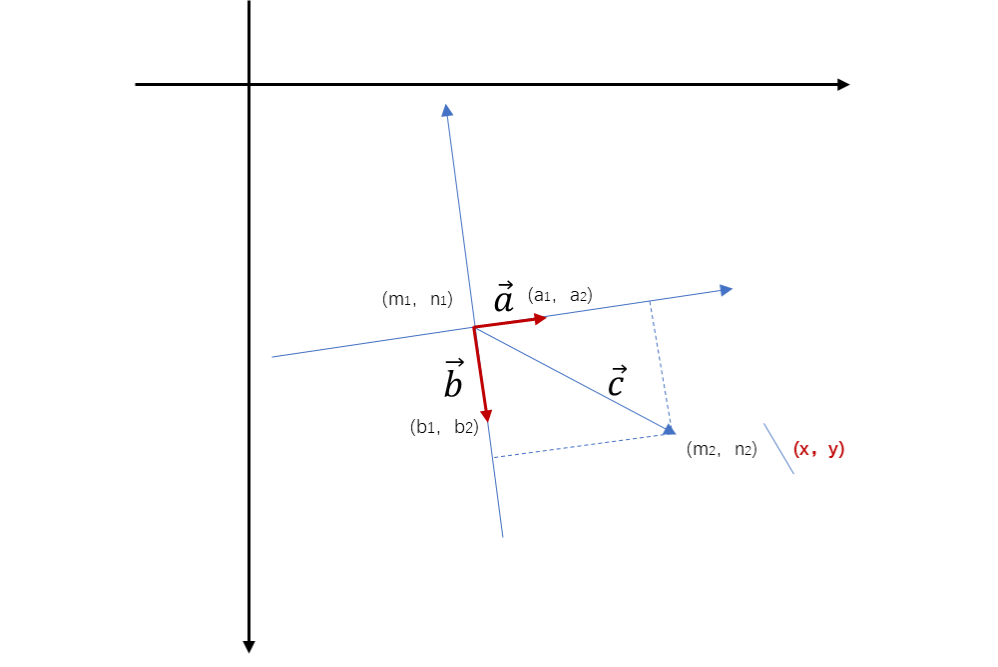
Coordinate system conversion, where the absolute coordinates (m_1_,n_1_), (m_2_, n_2_), (a_1_, a_2_), and (b_1_, b_2_) can be converted to the relative coordinates (0,0), (x,y), (1,0), and (0,1), respectively.

Assuming that the unit vector along the x axis in the relative coordinate system is vector *a* (*a*_1_, *a*_2_) and the unit vector along the y axis direction is vector *b* (*b*_1_, *b*_2_), we only need to compute (*x*,*y*), as shown in Figure 4. In Figure 4, the absolute coordinates (*m*_1_, *n*_1_), (*m*_2_, *n*_2_), (*a*_1_, *a*_2_), (*b*_1_, *b*_2_) can be converted to the relative coordinates (0, 0), (*x*, *y*), (1, 0), (0, 1), respectively.

The following relationships were satisfied between vectors:

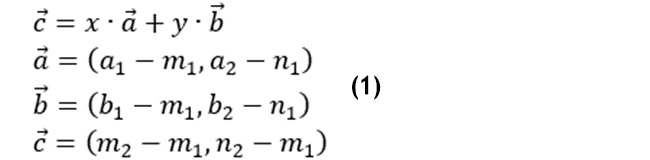



After calculation, the converted relative coordinates (*x*, *y*) were:

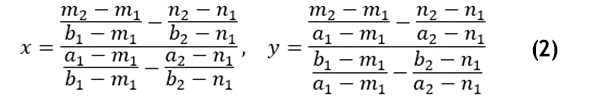



At this time, the value of (*x*, *y*) was the coordinate position in the relative coordinate system, which was converted by (*m*_2_, *n*_2_) in the absolute coordinate system.

### Key Facial Feature Extraction

Based on the facial expressions and tremors in patients with PD, we evaluated and extracted features from two main dimensions. One was the amplitude magnitude of the patient’s facial expression, which can be used to detect whether there is “mask face.” The other was judging the patient’s facial tremor through the face’s key points. When the tremor effects of some patients are obvious, head and elbow vibration will drive the head to perform regular tremors. For the 106 key points on the face, they were divided into two main parts, which were amplitude features of the facial expression and shaking features of the facial small muscle group.

#### Extraction of the Features of Facial Expression Amplitude

We defined a key point *z*’s position at the *i*th frame with a vector: ***p****_i_*_,_*_z_*=(*x_i_*_,_*_z_*,*y_i_*_,_*_z_*), in which *x_i_*_,_*_z_* and *y_i_*_,_*_z_* are the relative position’s horizontal coordinate and vertical coordinate, respectively. It is called the ***p***’s vector position.

For range *R*_max_, we defined the key point *z*’s range in the x-axis direction as *R*_x_, then:

*R*_x_max_*= x*_z_max_*– x*_z_min_ (**3**)

Similarly, the key point’s range in the y-axis direction was:

*R*_y_max_*= x*_z_max_*– x*_z_min_ (**4**)

Intuitively, covariance, Cov(X,Y), represents the expectation of the overall error of two variables, which can reflect the correlation of the patient’s expression amplitude changes in two directions, and it is calculated as follows:

Cov(X,Y) = *E*(XY) – *E*(X)*E*(Y) (**5**)

where *E*(X) and *E*(Y) are the expected values of *x* and *y*, respectively.

For absolute covariance, Cov(X_abs, Y_abs), we also calculated the covariance between the absolute coordinate X_abs, Y_abs. To some extent, it reflects the shaking of the human head.

#### Feature Extraction of the Tremor at Facial Key Points

Because patients with Parkinson disease patients cannot control their muscles like people without PD, the key points will tremble when they are trying to maintain facial expressions. To obtain the extent of tremor, we adopted absolute coordinates to measure the patient’s vibration. More specifically, we used ***p***_i,x_ = (x_iz_,y_iz_) to denote the position of the key point *z* at the *i*th frame. The Euclidean distance between the two locations was defined as:

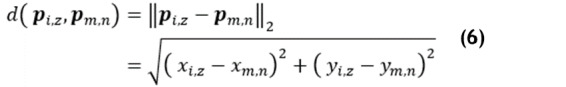



Moreover, when there were many position vectors, the set **P** was defined as:

**P** = {***p***_a(1,1), a(1,2)_, ***p***_a(2,1)a(2,2)_,…, ***p***_a(N,1)a(N,2)_} (**7**)

in which there exists *N* position vectors. Then, the average position of the set **P** was defined as:

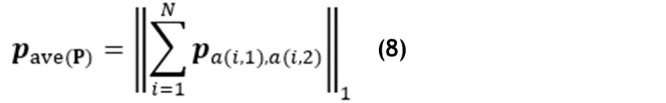



Jitter is a good measure of tremor [16]. However, it was created to calculate the tremor of a single signal. If we want to evaluate the positional Jitter of organ *z*, we take *N* frames of the organ, and the average position of the *N* frames is ***p***_ave(**P***_z_*). Then, positional jitter can be defined.

Jitter_abs is the relative Jitter of the key points (ie, the average absolute value of the positional distance between two adjacent frames).

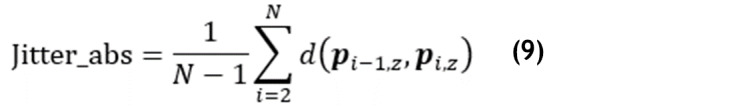



Jitter_PPQ5 represents the adjacent 5 points of Jitter at the key point (ie, the average absolute value of the difference between the position of a certain frame and the average position of the adjacent 5 frames).

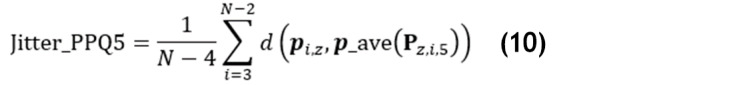



where **P***_z,i,_*_5_={***p****_i_*_-2_*_,z_*, ***p****_i_*_-1_*_,z_*, ***p****_i,z_*, ***p****_i_*_+1_*_,z_*, ***p****_i_*_+2_*_,z_*} denotes the average position of 5 adjacent frames (including the *i*-th frame) before and after the *i*-th frame of the key point *z*.

Jitter_rap represents the adjacent 3 points of jitter at the key point (ie, the average absolute value of the difference between the position of a certain frame and the average position of the adjacent 3 frames).

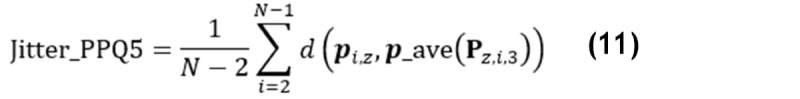



where **P***_z,i,_*_5_={***p****_i_*_-1_*_,z_*, ***p****_i,z_*, ***p****_i_*_+1_*_,z_*} is the average position of 3 frames (including the *i*th frame) before and after the *i*th frame of the key point *z*.

Jitter_ddp represents the difference between the adjacent 3 points of the key points’ jitter (ie, the difference between the distances of each adjacent 2 frames in the adjacent 3 frames). Then, the average absolute value was calculated as:

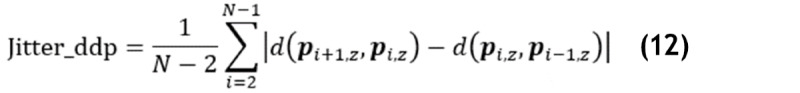



When we analyzed each patient’s key points, statistical analysis was performed on 106 key points. There were 8 features extracted for each key point, including 4 facial expression change amplitude features (*R_x_*__max_, *R_y_*__max_, Cov(X,Y), Cov(X_abs, Y_abs)) and 4 facial tremor features (Jitter, Jitter_PPQ5, Jitter_rap, Jitter_ddp). Since the collected data were not evaluated by a professional doctor using the UPDRS and we only knew whether the person had PD, we performed a diagnostic (classification) experiment. The final data statistics used in our experiment are shown in Table 2.

**Table 2:** Video data statistics.

Data statistics	Video data
Creation date	3/15/2018
Number of patients with Parkinson disease	33
Number of people without Parkinson disease	31
Number of records	176
Number of features	848
Task	Classification

## Results

In this paper, we performed a categorization task for facial expressions using widely used machine learning algorithms such as logistic regression (LR) [17], support vector machine (SVM) [18], decision tree [19], and random forest (RF) [20]. Table 3 shows that the methods based on facial key points can distinguish patients with PD from people without PD. The diagnostic result is relatively good.

**Table 3:** Experimental results of common machine learning algorithms.

Algorithm	Precision	Recall	F1 value
LR^a^	0.98	0.98	0.98
SVM^b^	0.99	0.99	0.99
DT^c^	0.93	0.93	0.93
RF^d^	0.98	0.98	0.98

^a^LR: logistic regression.

^b^SVM: support vector machine.

^c^DT: decision tree.

^d^RF: random forest.

In order to verify the significance of original features, we conducted hypothesis testing. In this experiment, the threshold value α, which is referred to as the level of significance, was set to .05 and .005 separately.

Table 4 shows the number of key points that reached significance for each of the 8 features types. At a *P* value <.05, all 106 key points were significant for all 4 tremor features at facial key points. At a *P* value <.005, all key points were significant for only 3 tremor features at facial key points. Therefore, we found that tremor features at facial key points may be more important than facial expression amplitude features.

**Table 4:** Number of points that reached significance for each feature type.

Feature name	Number of key points that reached significance	
	*P* value <.05	*P* value <.005
*R*_x_max_	83	69
*R*_y_max_	56	45
Cov(X, Y)	97	87
Cov(X_abs, Y_abs)	13	12
Jitter	106	106
Jitter_PPQ5	106	106
Jitter_rap	106	90
Jitter_ddp	106	106
Total	673	621

The least absolute shrinkage and selection operator (LASSO) is a linear regression method using L1 regularization [21]. LASSO can make part of the learned feature weights be 0, so it achieves the function of sparseness and feature selection simultaneously. In this experiment, we used LASSO to compress the features.

**Figure 5:**
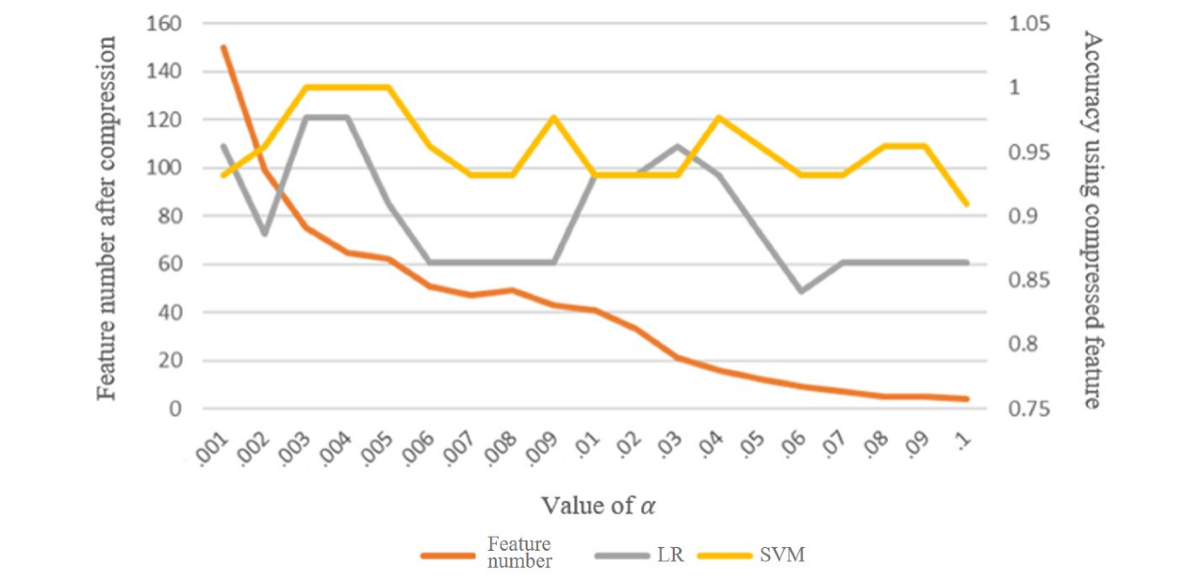
The effects of least absolute shrinkage and selection operator (LASSO) feature compression on logistic regression (LR) and support vector machine (SVM) models.

If a feature weight is nonzero in LASSO, this feature will be left; otherwise, this feature will be abandoned. The red line in Figure 5 shows that, as the coefficient α in LASSO increases, the number of features after compression decreases gradually. Then, we used the compressed features to perform the final classification task. As the number of features used in the training model decreases, the accuracy of the training set of the LR and SVM models changes (as shown by the gray and yellow lines, respectively). At an α value of .004, the two models (LR and SVM) had the highest prediction accuracy. However, as the original features were gradually reduced, the accuracy did not decrease linearly, which can prove the remaining features are more important than the discarded features.

**Figure 6:**
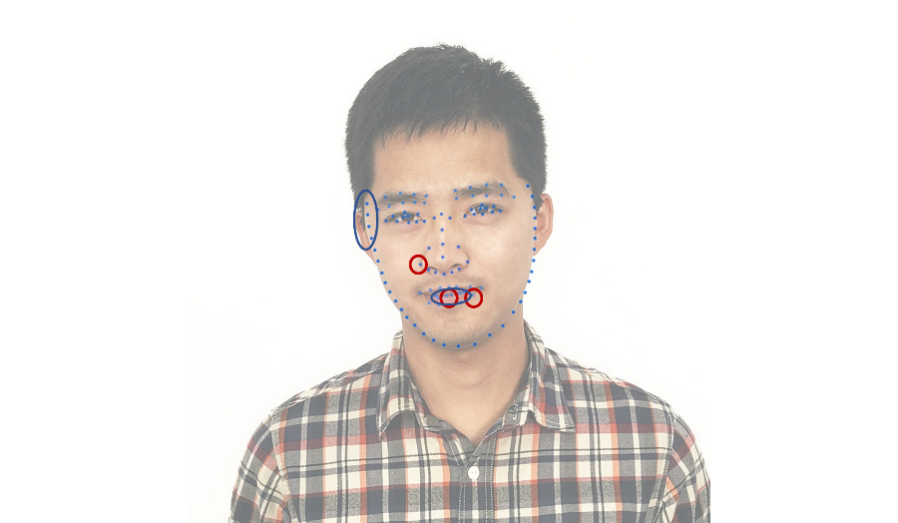
The key points that have a large influence on the classification result.

Then, we used LASSO with the best hyperparameters to obtain the most relevant features to the target (PD or not). At the same time, RF was used to sort out the importance of the features. These points are near the corner of the right eye and the lips on a human. In Figure 6, the features chosen by LASSO are marked with red circles, and the features chosen by RF are marked with blue ovals.

In this paper, we also utilized the advanced deep learning technique long short-term memory (LSTM) [22] to perform the classification. We converted the position changes of the key points in the x axis and y axis with time into time-series data. Then, we applied an order difference to the time series, that is, ∆*x_i_* = *x_i_*_-1_ - *x_i_*, ∆*y_i_* = *y_i_*_-1_ - *y_i_*. LSTM utilized these new time series in the model training. The results are shown in Table 5. The experimental results are relatively good too. Compared with the pure recurrent neural network technique [23], which has no effect on the classification, LSTM demonstrated it is more practical when dealing with sequential video data. Note that the facial video data used in this paper are limited. LSTM should be able to achieve better results if more data can be acquired.

**Table 5:** Experimental results of neural network models.

Algorithm	Precision	Recall	F1 value
LSTM^a^	0.86	0.66	0.75
RNN^b^	0.48	0.46	0.47

^a^LSTM: long short-term memory.

^b^RNN: recurrent neural network.

## Discussion

### Limitations

The amount of data that we collected was not sufficient. We hope to collect more data not only from patients with PD and people without PD but also from patients with other neurological diseases.

### Comparison With Prior Work

In the case of PD, to the best of our knowledge, there have only been a few software options, similar to ours, providing patients a convincing diagnosis using the facial recognition technique. These are compared to our work in Table 6.

**Table 6:** Comparison with a selection of prior work.

Work	Target and result	Data	Feature	Technology
Bandini et al [10]	Found PD^a^ patients have lower average facial expression movement distance; facial expression recognition for PD	17 PD patients, 17 healthy control subjects	Average distance of 49 facial key points in the facial expression movement	Face tracing, SVM^b^
Rajnoha et al [11]	Identified PD hypomimia by analyzing static facial images; less accurate compared with video-recording processing method.	50 PD patients, 50 healthy control subjects	128 facial measures (embedding) by CNN^c^	Face detector-based (HOG^d^), CNN, traditional classiﬁers (eg, random forests, XGBoost)
PARK^e^ framework by Langevin et al [12]	PARK instructs and guides users through 6 motor tasks and 1 audio task selected from MDS-UPDRS^f^ and records their performance by videos	127 PD patients, 127 healthy control subjects	Facial features: facial action units (AUs); motion features: motion magnitude metric of fingers and hands based on FFT^g^	OpenFace tool version 2, FFT
Our method	Proposed facial landmark features from videos to diagnose PD using facial expressions and achieved outstanding performance	33 PD patients, 31 healthy control subjects, 176 records	848 facial expression amplitude features and tremor features of facial key points; 65 features were left after feature compression	Face ++, traditional classiﬁers (LR^h^, SVM, DT^i^, RF^j^), LSTM^k^, LASSO^l^

^a^PD: Parkinson disease.

^b^SVM: support vector machine.

^c^CNN: convolutional neural network.

^d^HOG: histogram of oriented gradients.

^e^PARK: Parkinson's Analysis with Remote Kinetic-tasks.

^f^MDS-UPDRS: Movement Disorder Society Unified Parkinson Disease Rating Scale.

^g^FFT: fast fourier transform.

^h^LR: logistic regression.

^i^DT: decision tree.

^j^RF: random forest.

^k^LSTM: long short-term memory.

^l^LASSO: least absolute shrinkage and selection operator.

### Conclusions

In this paper, we established a diagnostic model for PD based on facial expressions. In the model, we formulated the diagnostic task into a classification problem. Then, we solved it by using algorithms from the area of traditional machine learning and the LSTM model from the field of deep learning research. When constructing video features, we conducted feature extraction according to the expression amplitude and degree of tremor. Using a fixed time interval and conversion of the coordinate system, the image was intercepted. This method converted the video into frame data. Further, the LSTM model was applied to the diagnosis of PD based on the generated time series data. Experimental results proved our model is effective and can be used as an efficient tool in PD diagnosis.

This article is a preliminary exploration of neurological diseases in the context of machine learning. The proposed method is designed to help patients get more comprehensive treatment and help doctors to understand the real-time dynamics of the disease. At the same time, it also aimed to relieve the problem of registering patients who have difficulty moving and to relieve the pressure on repeated patient diagnoses by doctors. With the development of science and technology, the introduction and application of artificial intelligence will bring more convenient and rapid diagnostic and treatment technologies.

## Abbreviations

CNN: convolutional neural network
DT: decision tree
FFT: fast fourier transform
HOG: histogram of oriented gradients
LASSO: least absolute shrinkage and selection operator
LR: logistic regression
LSTM: long short-term memory
MDS-UPDRS: Movement Disorder Society Unified Parkinson Disease Rating Scale
PARK: Parkinson's Analysis with Remote Kinetic-tasks
PD: Parkinson disease
RF: random forest
RNN: recurrent neural network
SVM: support vector machine
